# The effect of intergenerational support from children on loneliness among older adults-the moderating effect of internet usage and intergenerational distance

**DOI:** 10.3389/fpubh.2024.1330617

**Published:** 2024-04-08

**Authors:** Ruyi Huang, Rengui Gong, Qiong Deng, Yangming Hu

**Affiliations:** ^1^College of Public Administration and Law, Hunan Agricultural University, Changsha, China; ^2^College of Administration, Weifang Medical University, Weifang, China; ^3^School of Public Management and Law, Anhui University of Technology, Ma'anshan, China; ^4^College of Public Administration, Huazhong University of Science and Technology, Wuhan, China; ^5^College of Public Administration, Hunan Normal University, Changsha, China

**Keywords:** older adults, intergenerational support, loneliness, internet usage, intergenerational distance

## Abstract

**Objective:**

Loneliness is a key social and public health issue, mainly affecting the mental health of older adults. The article aimed to explore the influence of intergenerational support from children on loneliness among older adults. Meanwhile, the article also analyzed the moderating effects of internet usage and intergenerational distance in this process.

**Methods:**

Based on the data received from 2018 China Longitudinal Aging Social Survey (CLASS), the ordinary least square (OLS) regression model was used to analyze the influence of intergenerational support from children on loneliness among older adults. Furthermore, the Bootstrap method was used to test the moderating effect of internet usage and intergenerational distance on the relationship between intergenerational support from children on loneliness among older adults.

**Results:**

Baseline regression showed that economic support (*β* = −0.059, *p* < 0.001), caregiving support (*β* = −0.070, *p* < 0.001), and emotional support (*β* = −0.108, *p* < 0.001) from children can positively influence loneliness among older adults. Meanwhile, the results of the moderated effects analysis showed that internet usage and intergenerational distance moderates the relationship between caregiving support, emotional support from children and loneliness among older adults.

**Conclusion:**

The article demonstrates that family support, particularly intergenerational support from children plays a pivotal role in alleviating loneliness among older adults, so the government should further regulate the behavior of children’s alimony support, improve the digital infrastructure, these measures help to reduce loneliness among older adults and expand the depth and breadth of family care of older adults.

## 1 Introduction

Weiss (1) first described loneliness as an adverse emotional experience that occurs when there is a gap between expected and actual social relationships. Loneliness is a serious public health problem widely associated with aging and declining physical functioning. Surkalim et al. (2) showed that loneliness is common among older people. The prevalence of loneliness among older people from the Nordic countries to Eastern Europe is about 5.2 to 21.3%. The China 2019 Longitudinal Aging Social Survey also found that about 24.78% of older adults experience loneliness. Cacioppo et al. (3) showed that loneliness can lead to changes in the physiological, psychological, and social state of older adults, seriously affecting the physical and mental health of older adults and reducing their quality of life. For example, lonely older adults are more likely to develop depression and have a higher risk of developing cardiovascular disease (130% higher than those who are not lonely) (4).

Many studies have explored the relationship between social support and loneliness (5, 6). Social support is believed to come from different groups, such as friends, neighbors, and families. The intergenerational family support, especially from children, significantly impact the older adult’s physical and mental health due to the imperfect social security system of China’s society for old-age pension (7). Intergenerational support refers to the mutual support between parents and children, which involves reciprocity and transformation between children and parents (8). However, this research is limited to upward intergenerational support from children to their parents. Antonucci et al. (9) found that social support from their adult children significantly improves the psychological well-being and quality of life of older adults. Many scholars have also shown that loneliness is a predictive outcome of weak relationships between parents and adult children, such as lack of contact and support from adult children (10), and greater intergenerational conflict (11). However, some studies, such as Dean et al. (12), Adams et al. (13) have shown that receiving excessive intergenerational support from children can make older people aware of their aging and loss of control over their lives, thus raising loneliness (14). In china, the aging of the demographic structure may lead to smaller social networks and fewer young people who cannot meet the needs of older adults (15). Therefore, the impact of intergenerational support from children on loneliness among older adults may provide great academic and practical value in the new scenario.

Although many studies have assessed the relationship between intergenerational relationships and loneliness among older adults (16, 17), only a few studies have evaluated the extent of the relationship and the factors that influence the relationship. China has a differential pattern, where children play an important role in the lives of older adults. In addition, intergenerational relationship characteristics and structures have multidimensional attributes, including intergenerational contacts and interactions, affinity and distance, and intergenerational conflicts. However, it is unclear whether multidimensional intergenerational support is strongly associated with loneliness among older adults in the Chinese scenario. This study aimed to: (1) identify the relationship between different dimensions of children’s intergenerational support and loneliness among older adults; (2) analyse the heterogeneity of children’s intergenerational support across age groups, residential location, and marital status; (3) explore the mechanisms by which children’s intergenerational support affects loneliness among older adults.

## 2 Theoretical framework and assumptions

### 2.1 Social support theory

Raschke proposed that social support refers to the care and support that people feel from others. Social support is an interpersonal interaction that reduces the adverse effects of negative events on the individual and increases their self-confidence and sense of control over their lives. Dunér et al. (18) classified social support as formal support provided by governmental or non-governmental organizations and informal support provided by relatives, friends, neighbors, etc. Berkman et al. (19) explained the content of social support based on the following dimensions; instrumental support, informational support, and emotional support. Instrumental support is practical or tangible support (personal care, household chores, and financial support), while informational support refers to the transfer of information, including advice and recommendations. Emotional support is the sharing of happiness and sadness or the expression of care and concern. At the same time, studies have also assessed the relationship between social support and individual health. The main effect model has suggested that social support can improve mental health, regardless of whether an individual is stressed or not. Intergenerational support from children within the family validates this model. Specifically, Wu et al. (20) measured social support using financial and in-kind support for older adults from non-cohabiting children and found that higher financial support is significantly associated with lower loneliness among middle-aged and older parents, validating the main effects model. Guo et al. (21) also showed that children can reciprocate their parenting and express their care and regard to older adults through financial and caregiving support, which may reduce loneliness among older adults. They also showed that older adults get emotional exchange and communication through meeting and contacting their children to gain spiritual comfort, thus improving their mental health status. (22). The following hypothesis were proposed based on the social support theory and previous research results:

*H1*: Children’s comprehensive intergenerational support has a significant negative effect on loneliness among older adults. In this study, regression analyses were conducted based on three dimensions of intergenerational support, including financial support, caregiving support, and emotional support, to further develop three sub-hypotheses:

*H1a*: Children’s financial support will significantly diminish loneliness in older adults;

*H1b*: Children’s caregiving support will significantly diminish loneliness in older adults;

*H1c*: Children’s emotional support will significantly diminish loneliness in older adults;

However, the above effects may vary depending on older adult groups, such as different age groups, different residential locations, and different marital status. As a result, the following hypotheses were proposed:

*H2*: Intergenerational support has a significant heterogeneous effect on loneliness among different groups of older adults.

### 2.2 Internet usage, intergenerational distance, and loneliness in older adults

Some studies have assessed the mechanism by which intergenerational support from children affects loneliness in older adults from various perspectives, such as internet usage and intergenerational distance. Research in Israel found that internet usage improves socialization, thereby reducing loneliness (23). Moreover, a study in the United States also suggested that internet usage can reduce loneliness by keeping older people connected to their children and the community (24). Furthermore, studies conducted in the United States, Europe, and Asia-Pacific have found that social media use can effectively address social isolation and reduce depressive symptoms in older adults (25). Meanwhile, several studies have suggested that children’s residential distance from their parents and older adults’ residential patterns may directly or indirectly affect older adults’ loneliness and mental health. Besides, some studies have suggested that older people living with their children reduce their risk of getting lonely since their children take care of them. For example, Courtin and Avendano (26) showed that older people living with their children in Europe receive more life care and emotional support, thus reducing the risk of loneliness. Ten et al. (27) showed that low satisfaction of older people with their children and the absence of adult co-residing children are associated with more loneliness. Ekoh et al. (28) also indicated that epidemics limit rural older people’s proximity to their loved ones, leading to increased loneliness due to their dependence on intergenerational support.

Therefore, internet usage and intergenerational distance from children affect the relationship between intergenerational distance support from children and loneliness among older adults. As a result, the following hypotheses were proposed:

*H3*: Internet usage moderates the relationship between intergenerational support from children and loneliness among older adults.

*H4*: Intergenerational distance moderates the relationship between intergenerational support from children and loneliness among older adults.

## 3 Data and methods

### 3.1 Design study

The article is an original research study that explores the relationship between intergenerational support from children and loneliness among older adults, the design of this article includes the following components: (1) introduction; (2) research hypothesis; (3) data and methods; (4) results; (5) discussion; (6) conclusions and limitations. The data for this paper come from the China Longitudinal Aging Social Survey (CLASS) database, CLASS is designed by the Institute of gerontology and implemented by the china survey and data centre of Renmin University of China. During the fieldwork, each respondent who agreed to participate in the survey was asked to sign the informed consent, ethical approval for all the CLASS waves was granted from the institutional review board at Renmin University of China.

### 3.2 Data sources

**Figure 1:**
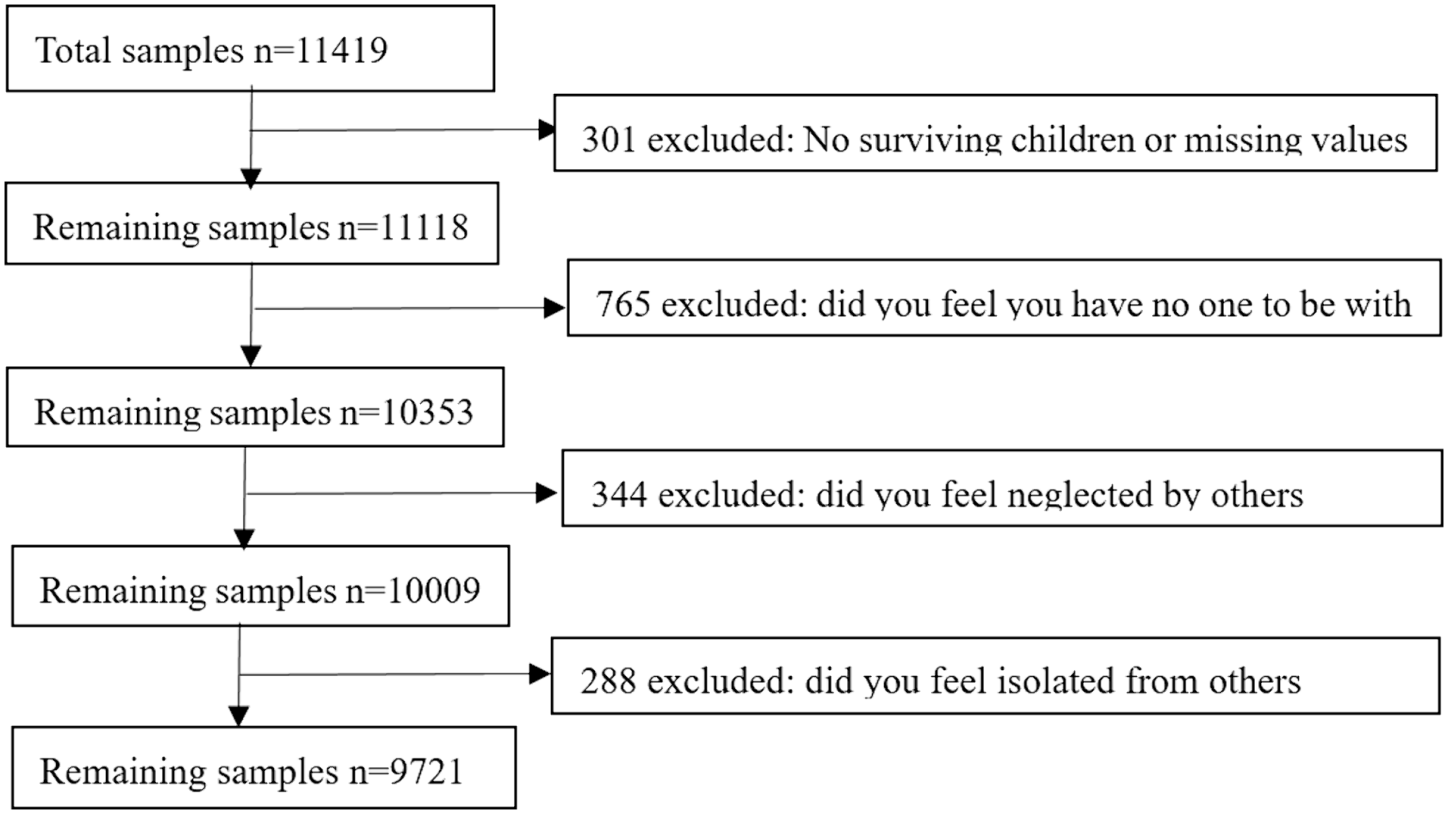
Flow chart of study samples.

Data were obtained from the 2018 China Longitudinal Aging Social Survey (CLASS). CLASS is a large-scale nationwide tracking survey, which aims to comprehensively understand the basic profile and socio-economic situation of older people in China (60 years old and above) to provide important references for the formulation of high-quality aging policies. A total of 11,418 individual questionnaires were obtained from 462 villages (residences) in 134 counties (districts) across China through stratified multi-stage probability sampling. Firstly, the samples without living children were deleted since this study focuses on the impact of intergenerational support of children on loneliness among the older adult. Secondly, the measurement of loneliness of older adults in the explanatory variables is based on the three loneliness measurement questions, accurate answers to the three questions are the basis for determining the loneliness of older adults, so respondents who did not answer the three questions accurately were excluded. Finally, a valid sample of 9,721 was obtained, See Figure 1.

### 3.3 Definition of variables

Assignments defines the core variables in this study, which include four main categories: explained variables, explanatory variables, control variables, and moderating variables.

#### 3.3.1 Explained variables

The explanatory variable of interest was loneliness among older adults. Loneliness was measured based on a three-item loneliness scale designed by Hughes, using the following questions: In the past week, did you feel that you have no one to be with? In the past week did you feel neglected by others? In the past week, did you feel isolated from others? The choices for each question were 1 = No, 2 = Sometimes, 3 = Often. The total loneliness score was obtained by adding the score of the answers to the three questions (total score ranged from 3 to 9), with higher scores indicating greater loneliness.

#### 3.3.2 Main explanatory variables

The scores of each entry of the explanatory variables were averaged by the number of children (after summing up the scores of all children) based on Buber’s categorization and calculation of intergenerational support (29). The economic support (children’s financial help to parents) was measured by whether the child has given the parent money, food, or gifts in the past 12 months (with a point rule ranging from 1 to 9). Caregiving support (life care, food, and clothing supply) was measured by how often the child has helped the parent with household chores in the past 12 months (with a point rule ranging from 1 to 5). Emotional support (children’s care and companionship to parents) was measured by how often the parent has met the child in the past 12 months (point scale ranging from 1 to 5).

#### 3.3.3 Control variables

The controls for demographic variables (gender, marital status, and educational level), social characteristics variables (household, income, and pension status), and health status variables (hypertension, self-assessed health status) were assessed as previously described.

#### 3.3.4 Moderating variables

The Pew Research Center (Pew Research Center) survey showed that the penetration rate of smartphones in China has reached 58%, with about 99.8% of the people using mobile phones to access the internet. In this study, use of smartphones was evaluated as previously described (30), using the following question; Have you used or not used a smartphone? (0 = No; 1 = Yes). The intergenerational distance was measured based on the nearest children’s usual place of residence to the older adults to examine the moderating role of intergenerational distance between intergenerational support and loneliness among older adults. The number of samples was noted for statistical purposes. Moreover, the eight options of children’s usual residence were divided into three categories, where the combination of this household and this village/neighborhood committee was regarded as living together (score; 1); the combination of this street/township and this district/county was regarded as living close to each other (score; 2), and the other place of residence was regarded as living far from each other (score; 3) (Table 1).

**Table 1:** Variable settings and assignments.

Dimension	Variable name	Definitions and assignments	Mean	SD
Explanatory variable	Loneliness	Based on the Hughes Scale, each item is scored on a scale of 1–3	4.50	1.59
Explained variable	Financial support	Cash equivalent of goods given to the older adult in the last 12 months, 1–9 points	4.15	1.82
	Caregiving support	Frequency of helping older adult of house-hold chores in the last 12 months, 1–5 points	2.73	1.23
	Emotional support	Frequency of meetings the older adult in the last 12 months, 1–5 points	2.89	0.66
Control variable	Gender	Male = 1 Female = 0	0.49	0.51
	Marital status	Having spouse = 1 No spouse = 0	0.70	0.46
	Educational level	From illiteracy to bachelor degree and above on a scale of 1–7	2.91	1.36
	Census register	Agricultural household = 1 Non-agricultural household = 0	0.56	0.50
	Income	Natural logarithm of personal income for the past 12 months	8.40	1.37
	Pension situation	Yes = 1 No = 0	0.42	0.49
	Self-Rated Health	Very healthy to very unhealthy on a scale of 1–5	2.67	0.91
Moderator variable	Internet usage	Yes = 1 No = 0	0.34	0.48
	Residential distance	Living together = 1 Near = 2 Far = 3	1.99	0.67

### 3.4 Research methodology

Loneliness of older adults was introduced into the model as the main explanatory variable during the model construction process since this study aimed to assess the impact of intergenerational support from children on the loneliness of the older adult and its mechanism of action. Notably, the OLS linear regression was used to measure loneliness in older adults (continuous variable), as shown in Eq.1:


(1) $$Y_{i}=\alpha_{0}+\alpha_{1}N_{1}+\alpha_{2}N_{2}+\alpha_{3}N_{3}+\beta_{i}X_{i}+\mu_{i}$$

𝑌_𝑖,_ 𝑁_1_,_2_, and 𝑁_3_ represent the loneliness, financial support, caregiving support, and emotional support, respectively. X_i_ represents the control variable. This study focused on the coefficients α_1_, α_2_, andα_3_, reflecting the effect of intergenerational support on loneliness in older adults. α_0,_ β_i,_ and 𝑢_i_ represent the intercept term, coefficient of the control variable, and random perturbation term, respectively.

## 4 Results

### 4.1 Descriptive analysis

From the sample data, it can be found that the mean value of the gender for the survey respondents is 0.49, the standard deviation is not large, showing that the ratio of men to women is basically the same, the mean value of the marital status is 0.70, indicating that the majority of the survey respondents have spouses; in terms of the level of education, the mean value is 2.91, the standard deviation is relatively small, we know that the educational level of survey respondents is not high, and concentrated in primary and lower secondary schools; the mean value of the household is 0.56, the proportion of agricultural households is higher than that of non-agricultural households.

### 4.2 Baseline regression analysis

In this paper, the changes in the coefficients of intergenerational support variables under different scenarios were explored based on multiple nested models to accurately measure the impact of children’s intergenerational support on loneliness among older adults in China’s special family relationship scenarios. Moreover, the four models established were tested for multiple linear relationships using multiple linear regression. The results showed that the variance inflation factor (VIF) of the four models was less than 10, indicating that the models established did not have multiple linear relationships and could be subjected to multiple linear regression models (I). The results after regression without adding any control variables showed that the coefficients of three forms of support were negative and significant at *p* < 0.001, indicating that children’s intergenerational support diminish loneliness among older adults, confirming hypothesis H_1_. The regression results of model (II) after demographic variables were added based on model (I) showed that: the intergenerational support, including children’s economic, caregiving, and emotional dimensions diminish loneliness in older adults. Compared with before the addition of control variables, the coefficients were slightly different, indicating the robustness of the regression results. For demographic variables, gender was not significantly associated with loneliness (*β* = 0.039, *p* > 0.05), inconsistent with other studies. For instance, Losada et al. (31) showed that loneliness is common among females than males due to the physiological disadvantages. However, marital status and educational level were significantly associated with loneliness. Low literacy levels and lack of spousal emotional support are associated with loneliness since such older people cannot have normal social life abilities. Moreover, such people can easily have psychological problems, such as loneliness (7, 32).

In models (III) and (IV), where social characteristics variables and health status variables were added, the coefficients were not significantly reduced, indicating that there is no significant linear relationship between the control variables and the independent variables, further confirming hypotheses H_1a_, H_1b_, and H_1c._ The insignificant effect of income on loneliness in older adults shows that income level cannot significantly influence loneliness and mental health of older adults, inconsistent with most studies (32). Table 2 shows the full study results.

**Table 2:** OLS estimates of the effect of intergenerational support on loneliness in older adults.

Variable	(I)	(II)	(III)	(IV)
Financial support	−0.059***(0.009)	−0.047*** (0.009)	−0.049*** (0.009)	−0.049** (0.009)
Caregiving support	−0.070*** (0.013)	−0.069*** (0.013)	−0.069** (0.014)	−0.067*** (0.014)
Emotional support	−0.108*** (0.024)	−0.099*** (0.024)	−0.098*** (0.024)	−0.101*** (0.024)
Gender		0.039 (0.033)	0.044 (0.033)	0.048 (0.033)
Marital status		−0.258*** (0.036)	−0.256*** (0.036)	−0.224***(0.034)
Educational level		−0.067*** (0.013)	−0.067*** (0.013)	−0.056***(0.013)
Census register			0.074 (0.040)	0.071 (0.039)
Income			−0.009 (0.013)	−0.007 (0.013)
Pension situation			0.068* (0.036)	−0.075* (0.036)
Self-rated health				−0.170*** (0.018)
Hypertension status				0.024 (0.034)
Constant	5.248*** (0.083)	5.520*** (0.089)	5.614*** (0.142)	5.075*** (0.150)
Observed	9,721	9,721	9,721	9,721
Adjustment of *R*^2^	0.011	0.021	0.022	0.031

***p < 0.001, **p < 0.01, *p < 0.05; standard errors in parentheses.

### 4.3 Heterogeneity analysis

In this paper, the heterogeneity of older adults with different ages, places of residence, and different marital statuses were analyzed through the OLS regression model to further validate the relationship between intergenerational support from children and loneliness of older adults and further improve the robustness of the regression model, Table 3 shows the full study results.

**Table 3:** Heterogeneity test table.

	(I)		(II)		(III)	
Variable	Young old	Oldest old	Village residence	Urban residence	Having spouse	No spouse
Financial support	−0.055***(0.011)	−0.050***(0.018)	−0.050***(0.014)	−0.045***(0.012)	0.055***(0.011)	−0.033(0.018)
Caregiving support	−0.058***(0.016)	−0.087***(0.026)	−0.070***(0.019)	−0.058** (0.029)	−0.042**(0.016)	−0.122***(0.025)
Emotional support	−0.112***(0.030)	−0.072***(0.041)	−0.131**(0.034)	−0.077**(0.029)	−0.096***(0.029)	−0.098*(0.026)
Control variable	Controlled					
Constant	4.472***(0.186)	4.638***(0.262)	5.037***(0.207)	3.639***(0.246)	4.624***(0.172)	4.680***(0.407)
Observed	6,804	2,917	5,470	4,251	6,811	2,910
Adjustment of *R*^2^	0.030	0.022	0.028	0.037	0.026	0.021

***p < 0.001, **p < 0.01, *p < 0.05; Standard errors in parentheses.

#### 4.3.1 Age

In this study, 60–75 years (excluding 75 years) and those older than 75 years were referred to as young old and oldest old based on the academic classification of the age stage of older adults. The regression results showed that intergenerational support had a different effect on loneliness in different age groups. Specifically, children’s emotional support significantly reduced loneliness in the younger seniors (*β* = −0.112, *p* < 0.001), while children’s caregiving significantly reduced loneliness in the older seniors (*β* = −0.087, *p* < 0.001).

#### 4.3.2 Residence (urban–rural)

Herein, those living in rural areas, towns outside county cities, and urban and rural assemblages were considered to be living in rural areas (score; 1). Moreover, those living in the central and marginal urban areas of cities and county cities were considered to be living in cities (score; 0). Grouped regressions showed that older people who live in the towns and villages (*β* = −0.131, *p* < 0.01) significantly rely on children’s emotional support than those in the urban areas. Furthermore, children’s caregiving support (*β* = −0.070, *p* < 0.001) significantly reduced loneliness among those living in urban regions compared with other regions (*β* = −0.058, *p* < 0.01).

#### 4.3.3 Marital status

Older adults with spouses were scored as 1, while those without spouses (widowed and divorced) were scored as 0. Subgroup regression showed that for older people without a spouse, the higher the demand for child support to achieve a decline in loneliness, especially caregiving support, whose coefficient was almost thrice that of the spousal older adults (*β* = −0.122, *p* < 0.001). However, child financial support was not significantly related to loneliness in older adults without spouses (*β* = −0.033, *p* > 0.05).

### 4.4 Endogeneity test

There were two main sources of endogeneity in the model (measurement error and selective bias). Certain factors may be related to both loneliness in old age and intergenerational support from children, such as relocation, which cannot be observed by the questionnaire. Therefore, the following should be conducted to avoid endogeneity.

#### 4.4.1 Replacement of the dependent variable

In this study, the dependent variable was replaced to further verify the robustness of the model, using the question “Do you feel lonely in the last week?“from the Glass database as a proxy indicator for loneliness. Ordered logistic regression results showed that although the estimated coefficients of children’s economic support, caregiving support, and emotional support changed, the direction and significance of the coefficients did not significantly change much, further verifying the robustness of the model, see Table 4 for details.

**Table 4:** Robustness test results based on variable substitution method.

Variable	Estimated value	Standard error	Wald value	*p* value
Financial support	−0.044	0.012	14.530	0.000***
Caregiving support	−0.036	0.017	4.503	0.034*
Emotional support	−0.117	0.032	13.126	0.000***
Control variable	Controlled			
Fit significance	0.000			

***p < 0.001, **p < 0.01, *p < 0.05.

#### 4.4.2 The propensity score matching (PSM) method

In this paper, Propensity Score Matching (PSM) 1:2 nearest neighbor matching method was used to control the bias caused by confounding factors on the model estimation and data results for testing the robustness of the results of OLS analysis. The results of the balance test after group matching according to children’s financial support (high or low) are shown in Table 5.The matching effect was detected by observing the error reduction value and t-test. The results showed that the standard deviation of all variables (except for the health self-assessment variable) was significantly reduced after re-matching (88.7–99.9%). Besides, the *p*-values of all variables were not significant after matching. Furthermore, the differences between the treatment group and the control group were substantially reduced, and the matching effect was better. In addition, the results of matching with caregiving support and emotional support also passed the balance test (results not shown).

**Table 5:** Error reduction before and after variable matching.

Variable	Pre/post match	Process group	Control group	Standard deviation	Deviation reduction	*T* value	*p* value
Educational level	Prematch	3.12	2.71	30.5	98.3	15.02	0.00
	After matching	3.12	3.11	0.5		0.25	0.80
Marital status	Prematch	1.27	1.34	−13.9	88.7	−6.86	0.00
	After matching	1.27	1.27	1.6		0.79	0.43
Census register	Prematch	1.64	1.43	35.6	99.9	17.51	0.00
	After matching	1.64	1.64	0.1		0.03	0.98
Self-rated health	Prematch	2.66	2.68	−2.0	−3.6	−0.97	0.33
	After matching	2.66	2.64	2.0		1.01	0.31
Hypertension status	Prematch	0.36	0.33	5.4	95.0	2.68	0.01
	After matching	0.36	0.36	0.3		0.13	0.90
Pension situation	Prematch	0.39	0.46	−13.3	97.4	−6.54	0.00
	After matching	0.39	0.39	0.3		0.17	0.87

Standard deviation, Deviation reduction presented in percentage.

The results of the ATT on the effect of intergenerational support from children on loneliness in old age under the nearest neighbor matching method are shown in Table 6. The coefficients of the impact of children’s financial support, caregiving support, and emotional support on loneliness in old age were 0.54, 0.59, and 0.43, respectively. The *T*-values were significant at the 5% significance level. Overall, the coefficients of the PSM regression results and the OLS regression results showed the same influence with slight differences. In conclusion, intergenerational support from children diminish loneliness in old age.

**Table 6:** Results of ATT estimation of intergenerational support on loneliness among older adults.

Variable	Estimated coefficient of ATT	Standard deviation	*T* value
Financial support	−0.54***	0.12	−4.52
Caregiving support	−0.59***	0.12	−5.13
Emotional support	−0.43**	0.22	−1.97

***p < 0.001, **p < 0.01, *p < 0.05.

### 4.5 Analysis of impact mechanisms

We use the Bootstrap method to test the moderating effect of internet usage, children’s intergenerational residential distance on the relationship between children’s intergenerational support and loneliness of older adults, in Process plug-in with a 95% confidence interval, 5,000 as the sample size, and the model sequence number was chosen as Model 1. Initially, the independent variables including children’s economic support, caregiving support, and emotional support, along with the moderating variables concerning the Internet usage of older adults and the intergenerational residential distance of children, were centered. Subsequently, hierarchical regression analyses were employed. This process involved controlling for covariates such as gender, education level, income, and physical health status. The subsequent steps entailed the gradual addition of intergenerational support, Internet usage by older adults, children’s intergenerational residential distance, and their respective interaction terms with children’s intergenerational support. The aim was to monitor the changes in significance for each variable, see Table 7 for details.

**Table 7:** Analysis of the moderating effects of Internet usage and intergenerational residential distance in the impact of intergenerational support on loneliness among older adults.

Variable	(I)	(II)	(III)
Financial support	−0.050** (0.009)	−0.050*** (0.009)	−0.059*** (0.009)
Caregiving support	−0.066*** (0.013)	−0.072*** (0.013)	−0.080*** (0.013)
Emotional support	−0.100*** (0.024)	−0.130*** (0.025)	−0.118*** (0.024)
Financial support × Internet usage		−0.024 (0.020)	
Caregiving support × Internet usage		0.137*** (0.032)	
Emotional support × Internet usage		−0.148* (0.070)	
Financial support × intergenerational distance			0.022 (0.013)
Caregiving support × intergenerational distance			0.070*** (0.020)
Emotional support × intergenerational distance			0.074*(0.034)
Control variable	Controlled	Controlled	Controlled

***p < 0.001, **p < 0.01, *p < 0.05.

Model (I) refers to the regression result that does not include any interaction term, and model (II) puts in the interaction term of children’s intergenerational support and Internet usage. Analysis of the regression results indicated that the moderating effect of Internet usage of older adults on the relationship between children’s economic support and loneliness of older adults is not significant (*β* = 3.825, *p* > 0.05). However, it has a moderating effect on the relationship between children’s emotional support and loneliness of older adults (*β* = 3.920, *p* < 0.05), indicating that the emotional support provide by children for older adults with high Internet usage can reduce loneliness among older adults. Results showed that the moderating effect for the relationship between children’s caregiving support and loneliness among older adults was significant (*β* = 3.801, *p* < 0.001). The addition of the Internet usage variable, weakened the negative effect of child care support and loneliness of older adults. Its impact on older people with low internet usage became more pronounced when there was a reduction in the caregiving support from children.

**Figure 2:**
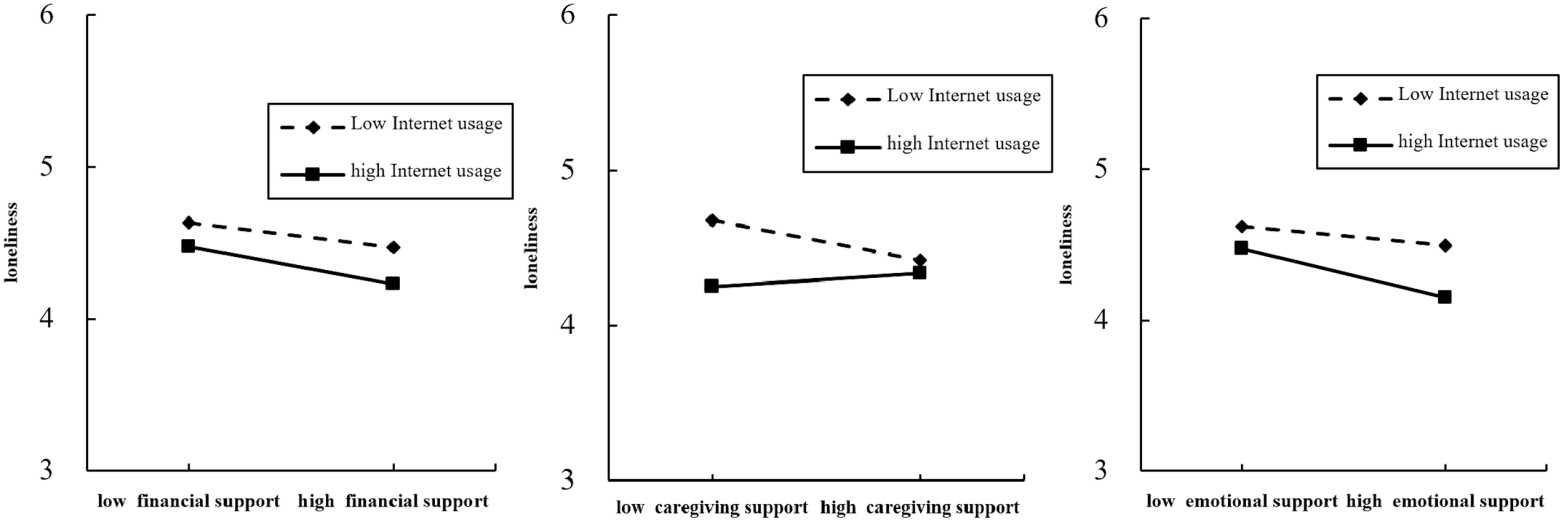
Moderating role of internet usage between intergenerational support and loneliness among the older adult.

**Figure 3:**
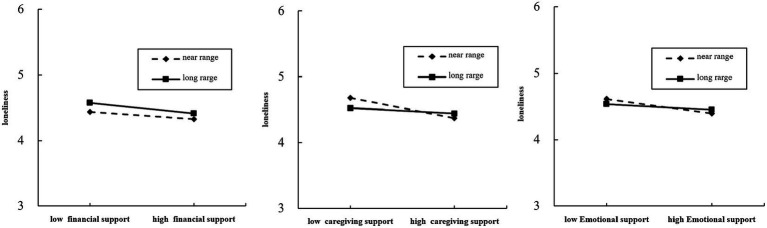
Moderating role of intergenerational distance between intergenerational support and loneliness among the older adult.

Model (II) was put into the interaction term between child intergenerational support and child intergenerational residential distance, and the regression results revealed that the moderating effect of child intergenerational residential distance on the relationship between child financial support and loneliness among older adults was not significant (*β* = 3.865, *p* > 0.05), but the moderating effect of child intergenerational residential distance on the relationship between child care support and loneliness among older adults was highly significant (*β* = 3.872, *p* < 0.001), indicating that the addition of the intergenerational residential distance variable weakened the negative effect of the influence of children’s caregiving support and loneliness of older adults. This was indicated by the fact that when children’s caregiving support increased, older people living with their children or in close proximity to them exhibited a significant decrease in loneliness The moderating effect of children’s intergenerational residential distance on the relationship between children’s emotional support and loneliness of older adults was significant (*β* = 4.013, *p* < 0.05). Given that children’s emotional support and loneliness of older adults showed a negative correlation, the moderating effect of the children’s residential distance had a significant inhibitory effect on the relationship between emotional support and loneliness. To further validate the moderating effect of Internet usage and children’s residential distance on the relationship between children’s intergenerational support and loneliness of older adults, we developed a simple slope analysis graph (Figures 2, 3) with one standard deviation above and one standard deviation below. The varying slopes of the curves depict the extent and direction of the moderating influences of internet usage and intergenerational distance on the correlation between economic support, caregiving support, emotional support, and the loneliness experienced by older adults.

## 5 Discussion

Intergenerational support from children can positively reduce loneliness among older people. Although some scholars, Guo et al. (21) and Djundeva et al. (33), have argued that excessive financial and care may lead to older parents becoming dependent on their adult children, which can be detrimental to mental health and require them to bear the stigma of being a “burden” to their children. Nonetheless, our findings align with the consensus among scholars, affirming the positive impact of children’s financial, caregiving, and emotional support on alleviating the loneliness experienced by older adults. This is underscored by the consistently negative estimated coefficients, all of which yield a *p*-value below 0.05. However, it is worth noting that there is a significant variability in the impact of the three dimensions of children’s intergenerational support on the loneliness of older adults. Sun et al. (34) argued that compared to financial support and caregiving support, emotional support can potentially promote the mental health of older adults. In this analysis, we showed that without adding any control variables, children’s emotional support has the highest importance among the three dimensions (*β* = −0.108), implying that each level of children’s emotional support for older adults can reduce the loneliness score of older adults by 0.108. Regarding its role and value is almost double that of financial support (*β* = −0.059). The regression results after adding control variables still indicate that the estimated coefficient of children’s emotional support exceeds that of the other two. This result may be explained by Liu et al. (35), who have argued that the improvement of pension insurance, medical insurance and other security systems implemented by the Chinese government in recent years has had a “crowding out” effect on intergenerational financial support, the majority of older adults people in China can rely on their own incomes to satisfy their basic daily needs. In addition, they found that the importance of emotional communication between children and their parents has gradually been highlighted, Children’s multi-frequency communication and comfort with parents through various means can effectively alleviate the loneliness experienced by older adults due to widowhood, illness and other reasons. This further confirms the conclusion that in-person interactions with non-resident children reduce loneliness among older individuals.

The relationship between intergenerational support for children and loneliness among Chinese older adults varies among different groups. Jiang et al. (36) observed a significant decline in the physical and physiological functions of older adults, As a result, they are susceptible to the risk of incapacitation and rely more on their children’s intergenerational support, resulting in more substantial caregiving support from their children. This conclusion is confirmed by the results of the textual study, which found that emotional support from children was more important for the young old and caregiving support was more important for the oldest old.

Residential environment (rural, small town vs. urban) is associated with loneliness, and the regression results in this paper demonstrate that high frequency of contact from children improves physical and mental health of rural older adults (*β* = −0.131, *p* = 0.000), but has a smaller relative effect on urban older adults (*β* = −0.077, *p* = 0.024), which is fully consistent with the findings of Chinese scholars (37). Paúl et al. (38) postulated that people living in urban environments have higher levels of loneliness than those in rural areas. However, in this paper, we found that older people living in rural areas have a decreased sense of loneliness and need more intergenerational support from their children. The disparity mentioned above primarily stems from the persisting urban–rural divide in China. The majority of rural areas continue to lag significantly behind urban areas in terms of infrastructure, healthcare facilities, recreational amenities, and more. This situation imposes considerable inconvenience on the lives of older individuals, especially those with compromised physical conditions, so they are in critical need of enhanced care and emotional support from their children to alleviate the feelings of helplessness and the sense of loss resulting from the challenges of daily life. Marital status has a significant effect on the relationship between intergenerational support for children and loneliness among older adults. This may be ascribed to the fact that the presence or absence of a spouse affects loneliness among older adults; those who have a spouse having lower levels of loneliness compared to others (39). Zhang et al. (40) also revealed that among family members, spouses are the preferred recipients of help, followed by children. For older adults who live alone without a spouse or whose spouses have died, their children become the most important part of their daily life. This finding underscores the importance of children providing increased care and emotional support to older adults who live alone without a spouse, as it proves to be particularly effective in alleviating loneliness in this demographic.

The moderating effect of Internet usage on the association between children’s financial support and loneliness among older people is not significant, and the relationship between caregiving support and loneliness among older people is highly significant. The core explanation for this may be due to the fact that for child financial support, the value of its effect on the reduction of loneliness in older people is strongly related to the level of children’s income, and the importance that older individuals place on money. Financial payments from children to their parents can also be made in kind, in cash, by transfer, and through other forms. None of this has much to do with whether older people use the internet or not.

In the current context of widespread smartphone usage in China, older adults are now able to independently engage in activities such as shopping, seeking medical treatment, and traveling through the use of smartphones. The present results show that the use of the Internet provides convenience to older adults and significantly improves their subjective sense of well-being, the use of the Internet allows older adults to avoid relying entirely on their children for care in their daily lives, thus weakening the value of children’s care support in the process of reducing loneliness. The Internet provides a degree of independence for older adults, reducing their reliance on in-person care from their children. This, in turn, moderates the impact of child caregiving support on alleviating loneliness among the older adult, with the level of the older adult’s Internet usage playing a significant role. Although some scholars believe that Internet usage can reduce loneliness by increasing intergenerational interactions between older adults and their families, our findings suggest that Internet usage is currently not an effective substitute for the emotional support provided by children for older adults, and that it is not a protective factor against loneliness in older adults. For this point, some scholars have also obtained similar results. Fang et al. (41) investigated 738 older adults aged 60 and above in Hong Kong, China, showed that the higher the frequency of Internet usage, the higher the psychological stress and the higher the level of loneliness among older adults. Baker and Algorta (42) argued that negative interactions and social comparisons on the Internet may exacerbate the Rebellion of certain Internet users against mainstream social values, exacerbate loneliness and alienation, cause Internet addiction, apathy and fear. In summary, while Internet use by older adults provides certain conveniences, it cannot substitute the essential emotional and spiritual support derived from interactions with their children. On the contrary, the potential drawbacks of excessive Internet use in this demographic should be mitigated through regular phone conversations, frequent visits, and the provision of ample care and comfort by their children.

The moderating effect of intergenerational residential distance of children on the relationship between intergenerational support for children and loneliness among older adults exhibits high variability. Given that the current financial support of children to older adults can be implemented through various channels and forms, it is not dependent on children living with older adults or living in close proximity, therefore the intergenerational residential distance is not effective in moderating the relationship between children’s financial support and loneliness in older adults. Gruijters (43) found that children residing with or in close proximity to older adults find it more convenient to provide them with essential care like food, household chores, and nursing. This paper’s findings further validate the substantial impact of intergenerational residential distance on the correlation between children’s caregiving support and the loneliness experienced by older adults. In contrast to children living nearby, those residing in other provinces or abroad may encounter challenges in regularly attending to their parents, especially when they are in declining health. This can hinder their ability to meet their parents’ caregiving needs effectively. Connelly et al. (44) argues that the frequency of meeting and contact between older adults and their children will be higher under the conditions of proximity living, which reduces the feeling of loneliness among older adults., Even though the children living in close proximity do not frequently meet older adults due to their work, family and other reasons, they can quickly and timely reach them when older adults suffer from illnesses and other emergencies affecting their lives, which may improve the quality of life of older adults by improving the accessibility of healthcare services and other channels. Therefore, it can enhance their health (45, 46). The present findings better support the above conclusion.

## 6 Conclusions and limitations

The article draws a series of conclusions that may be valuable for future research. Intergenerational support from children plays a pivotal role in alleviating loneliness among older adults, in this process the moderating effect of internet usage and intergenerational residential distance between intergenerational support for children and loneliness among older adults exhibits high variability. While there are some limitations in this study, for example the data included in this paper are cross-sectional data rather than panel data, and hence it may not accurately reflect the dynamic changes of loneliness in older adults and children support. The quantification standard of loneliness in old age may be simple and not sufficiently comprehensive, more longitudinal studies should be conducted in future to explore changes in the experience of loneliness and related determinants of loneliness among older adults over time.

Finally, we suggest that the government need to further regulate the behavior of children’s alimony support for their parents through publicity and education, laws and regulations. In addition, the government should develop policy measures to address the loneliness of older adults in the rural areas, older adults in the older age groups, and older adults who are widowed. The study also shows that Internet usage and intergenerational living distance play a moderating role in the relationship between intergenerational support for children and loneliness among older adults. In this way, the government can help older adults to use the Internet appropriately and reasonably by improving the digital infrastructure and establishing a good feedback mechanism. In future, we will expand and strengthen the county economy and promote the revitalization of the countryside to increase the number of young and middle-aged people in towns and villages where they can live and work locally. This would facilitate more effective intergenerational support for children, ultimately leading to a reduction in the sense of loneliness experienced by older adults.

## Data availability statement

The original contributions presented in the study are included in the article/supplementary material, further inquiries can be directed to the corresponding author.

## Ethics statement

Written informed consent was obtained from the individual(s) for the publication of any potentially identifiable images or data included in this article.

## Author contributions

RH: Data curation, Software, Writing – original draft, Formal analysis, Methodology, Writing – review & editing. RG: Writing – review & editing, Formal analysis, Resources. QD: Writing – review & editing, Data curation. YH: Writing – review & editing, Funding acquisition, Supervision.
